# Retraction Note: Lateral palatal foramina do not indicate baleen in fossil whales

**DOI:** 10.1038/s41598-025-88714-w

**Published:** 2025-02-11

**Authors:** Carlos Mauricio Peredo, Nicholas D. Pyenson, Mark D. Uhen

**Affiliations:** 1 Department of Earth and Environmental Science, University of Michigan, Ann Arbor, MI USA; 2 Department of Marine Biology, Texas A&M University, Galveston, TX USA; 3 Department of Paleobiology, National Museum of Natural History, Washington, DC USA; 4 Departments of Mammalogy and Paleontology, Burke Museum of Natural History and Culture, Seattle, WA USA; 5 Department of Atmospheric, Oceanic, and Earth Sciences, George Mason University, Fairfax, VA USA

Retraction of: *Scientific Reports* 10.1038/s41598-022-15684-8, published online 06 July 2022

The Editors have retracted this Article. Following publication concerns were raised by Ekdale et al.^1^ about the reproducibility of the methods used and analysis of the data, including the undercounting of the number of foramina in mysticetes and the inflated number in terrestrial artiodactyls. Post-publication peer review has confirmed the validity of these concerns. The Editors therefore no longer have confidence in the conclusions presented.

The authors disagree with this retraction.
